# Supplementary material for: KF4 anti-CELA1 Antibody and Purified α1-Antitrypsin Have Similar but Not Additive Efficacy in Preventing Emphysema in Murine α1-Antitrypsin Deficiency
Source: bioRxiv. 2024 May 10:2024.05.07.592994. Preprint. [Version 1] doi: 10.1101/2024.05.07.592994 (PMC11100728; doi:10.1101/2024.05.07.592994)
Supplement: Supplement 1 [file media-1.pdf]

Histological Signs of Liver Injury

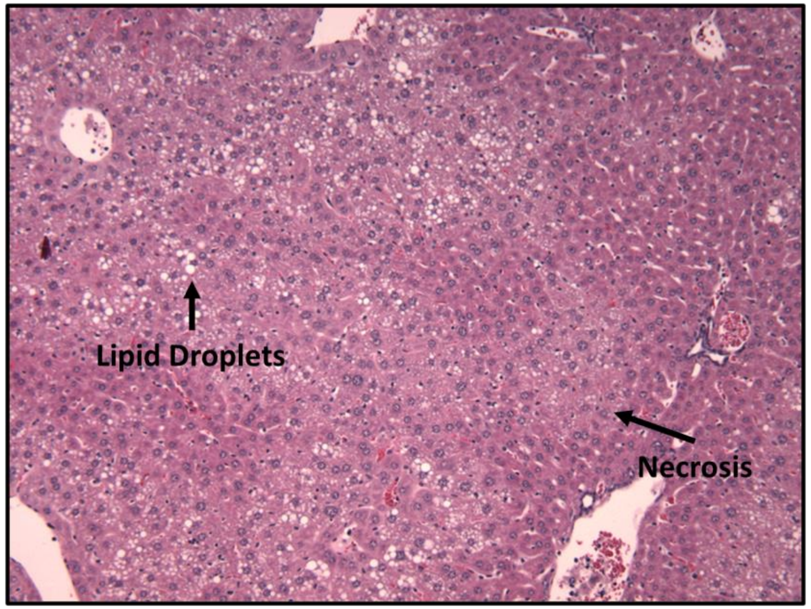

Healthy Liver Tissue

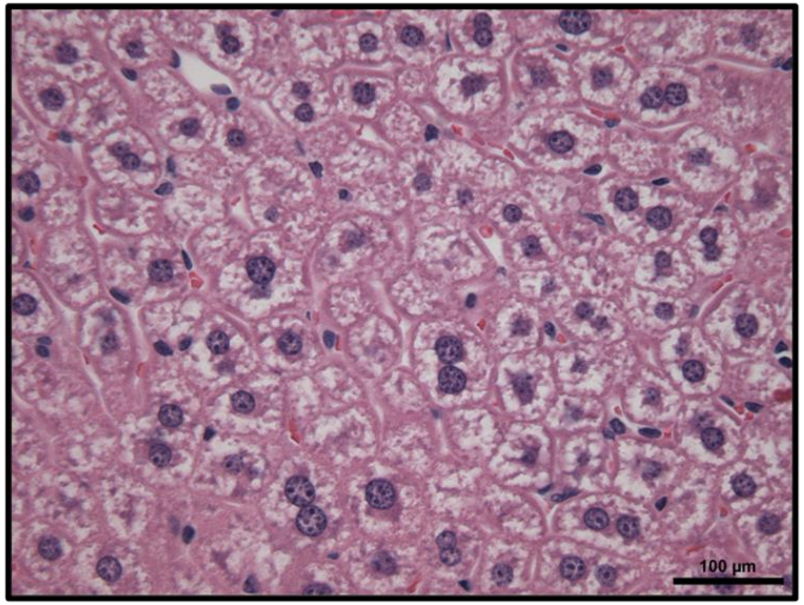

Septic Liver Tissue

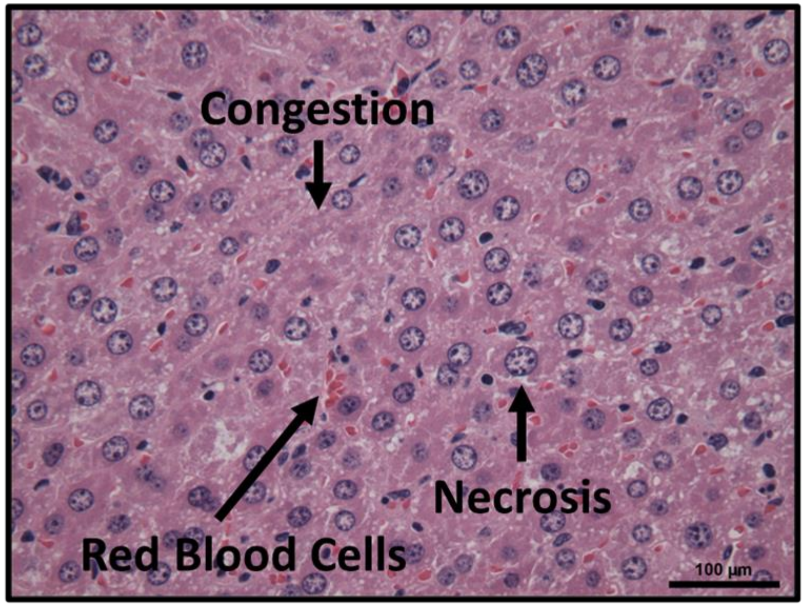

Scores
0 = no injury
1 = minimal (0-25%of the section)
2 = mild (25-50%)
3 = significant (50-75%)
4 = severe (more than 75%)

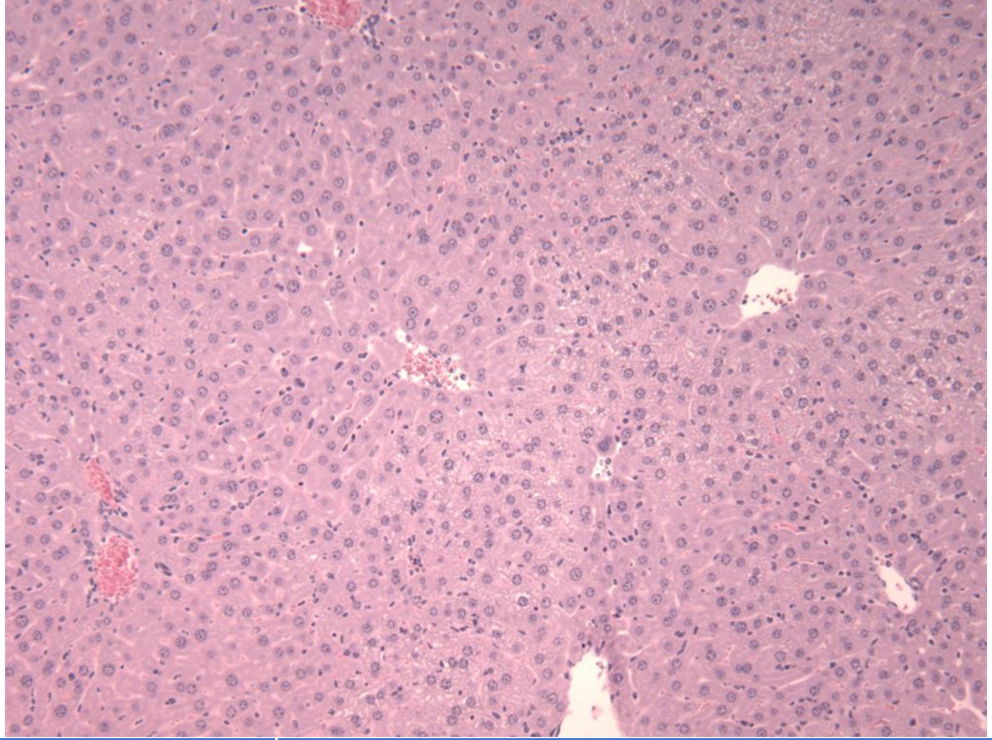

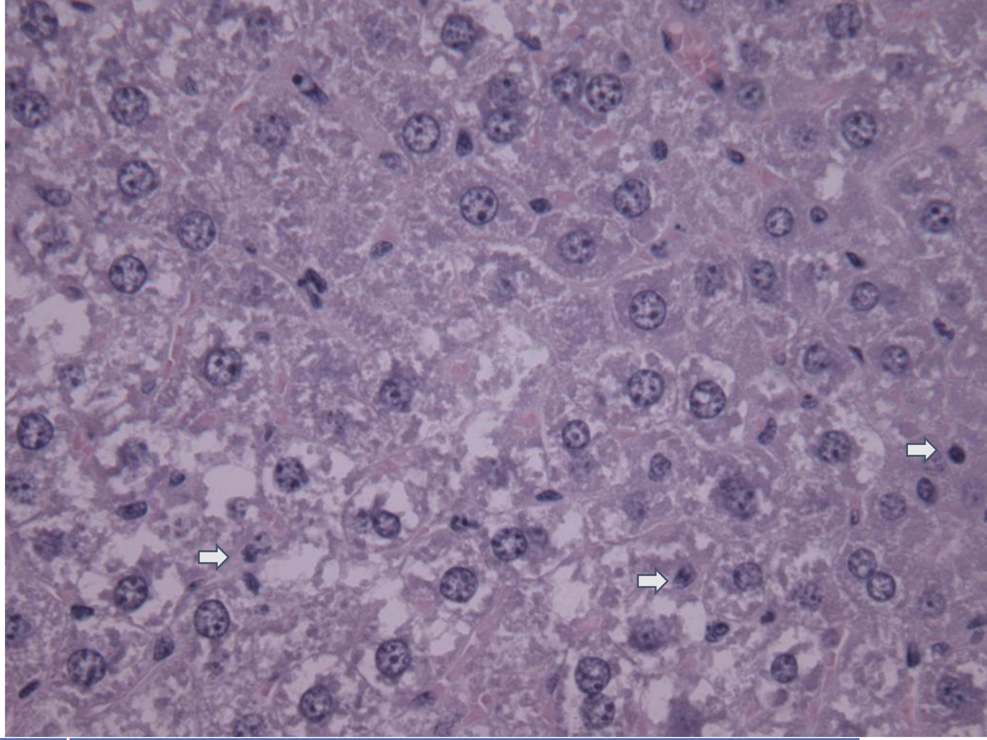

	10x	40x
Sinusoid congestion and/or edema (10X)	3	
Infiltration of red blood and inflammatory cells (10X)	2	
Presence of lipid droplets or vacuoles (10 & 40X)	0	0
Necrosis (10 & 40X)	3	3
Total		

Scores
0 = no injury
1 = minimal (0-25%of the section)
2 = mild (25-50%)
3 = significant (50-75%)
4 = severe (more than 75%)

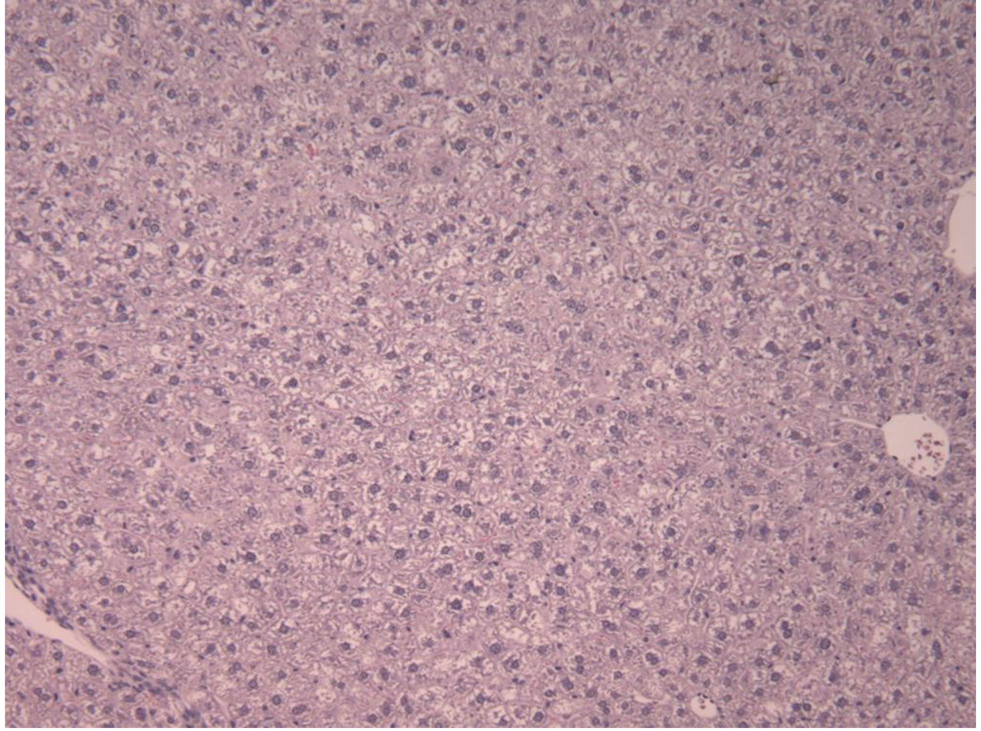

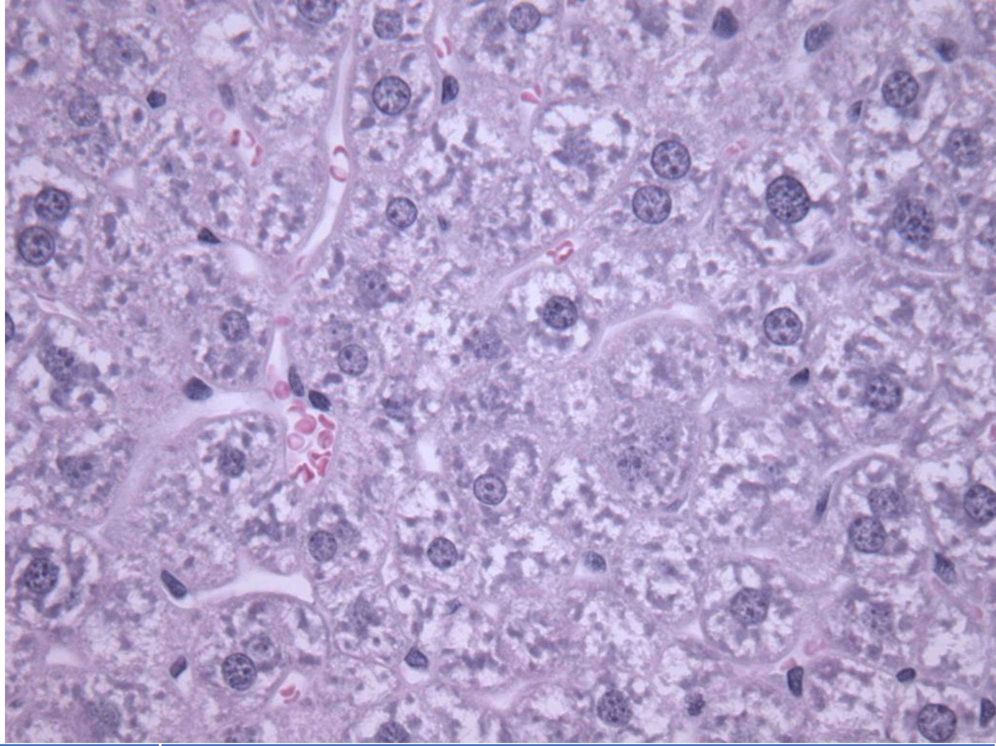

	10x	40x
Sinusoid congestion and/or edema (10X)	0	
Infiltration of red blood and inflammatory cells (10X)	0	
Presence of lipid droplets or vacuoles (10 & 40X)	0	0
Necrosis (10 & 40X)	1	1
Total		

Scores
0 = no injury
1 = minimal (0-25%of the section)
2 = mild (25-50%)
3 = significant (50-75%)
4 = severe (more than 75%)

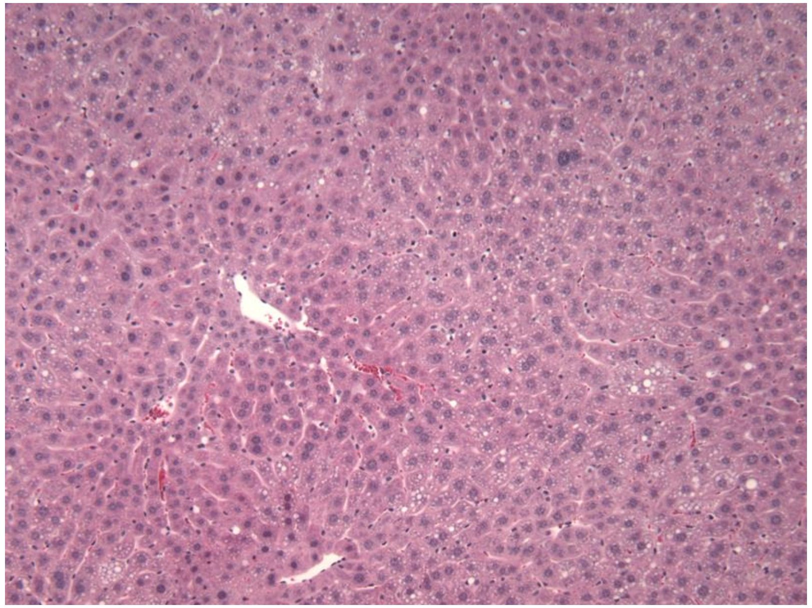

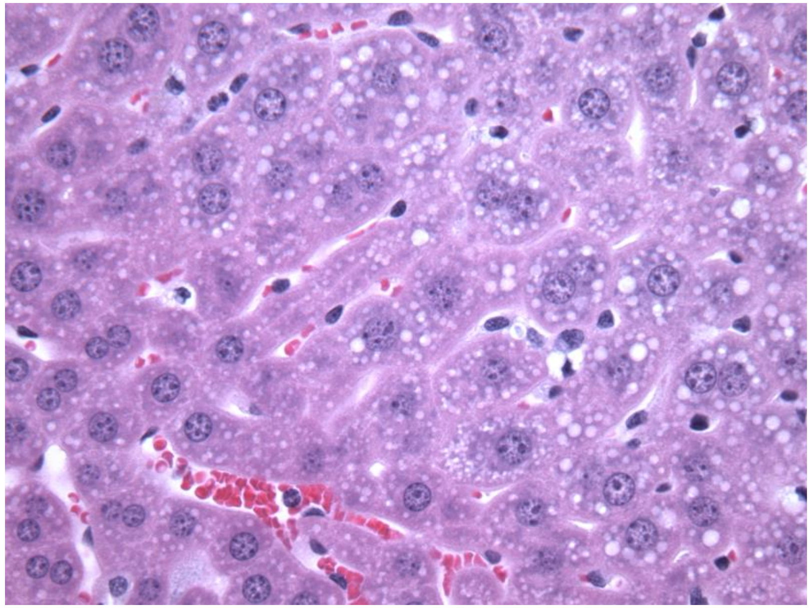

	10x	40x
Sinusoid congestion and/or edema (10X)	3	
Infiltration of red blood and inflammatory cells (10X)	3	
Presence of lipid droplets or vacuoles (10 & 40X)	3	4
Necrosis (10 & 40X)	3	3
Total		
