# Supplementary material for: KF4 anti-CELA1 Antibody and Purified α1-Antitrypsin Have Similar but Not Additive Efficacy in Preventing Emphysema in Murine α1-Antitrypsin Deficiency
Source: bioRxiv. 2024 May 10:2024.05.07.592994. Preprint. [Version 1] doi: 10.1101/2024.05.07.592994 (PMC11100728; doi:10.1101/2024.05.07.592994)
Supplement: Supplement 2 [file media-2.pdf]

Histological signs of kidney injury-10X

Healthy Kidney Tissue

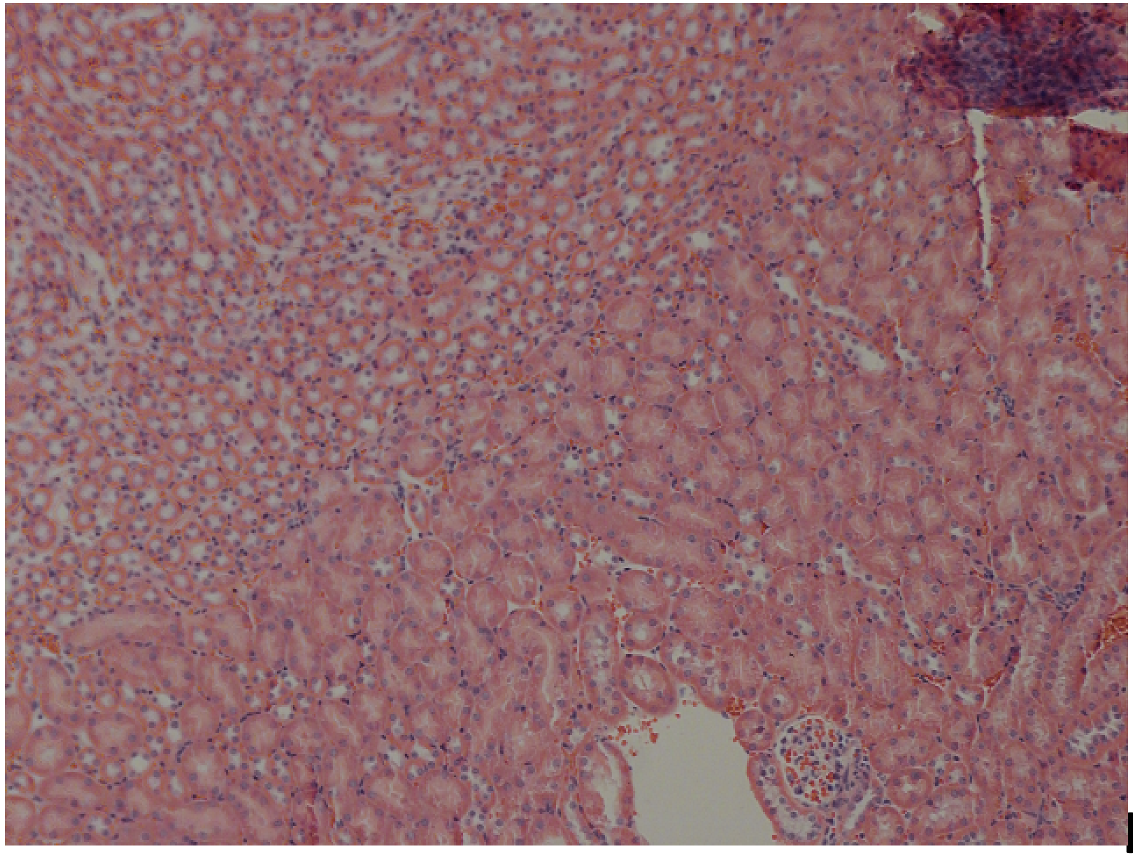

I/R Kidney Tissue

Leukocytes

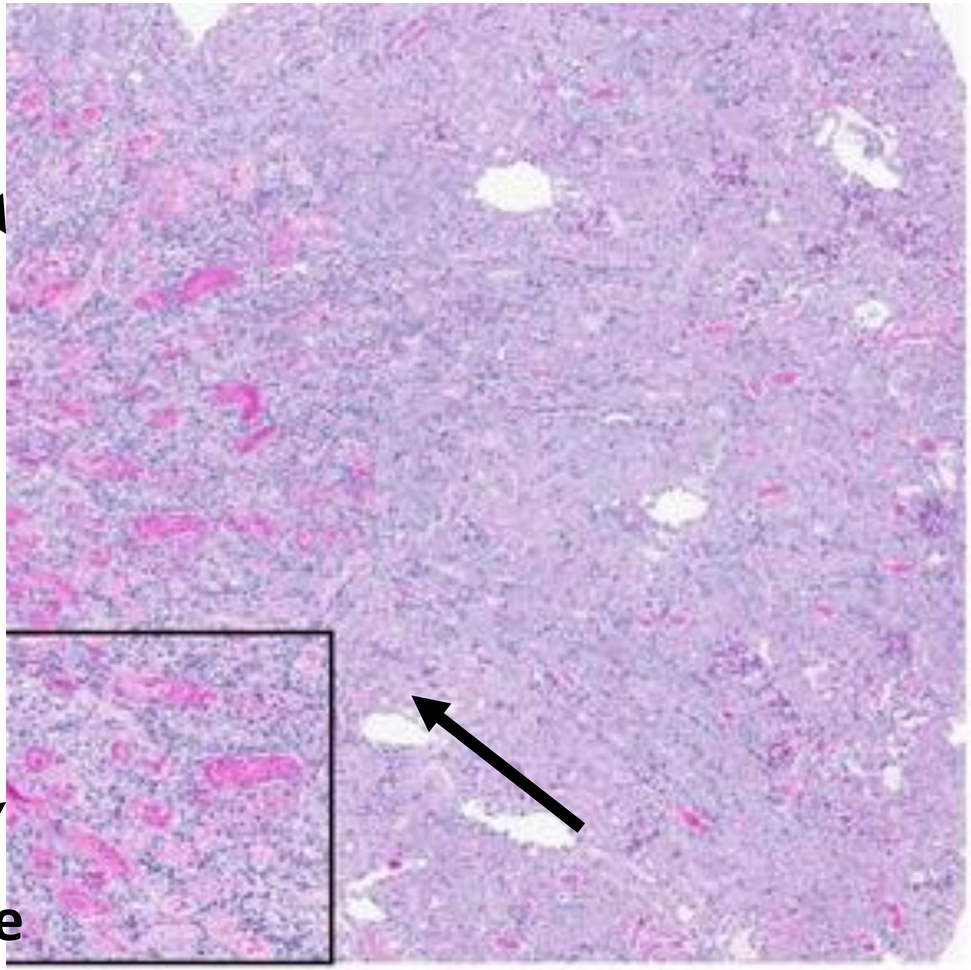

Hemorrhage

Histological signs of kidney injury-40X

Healthy Kidney Tissue

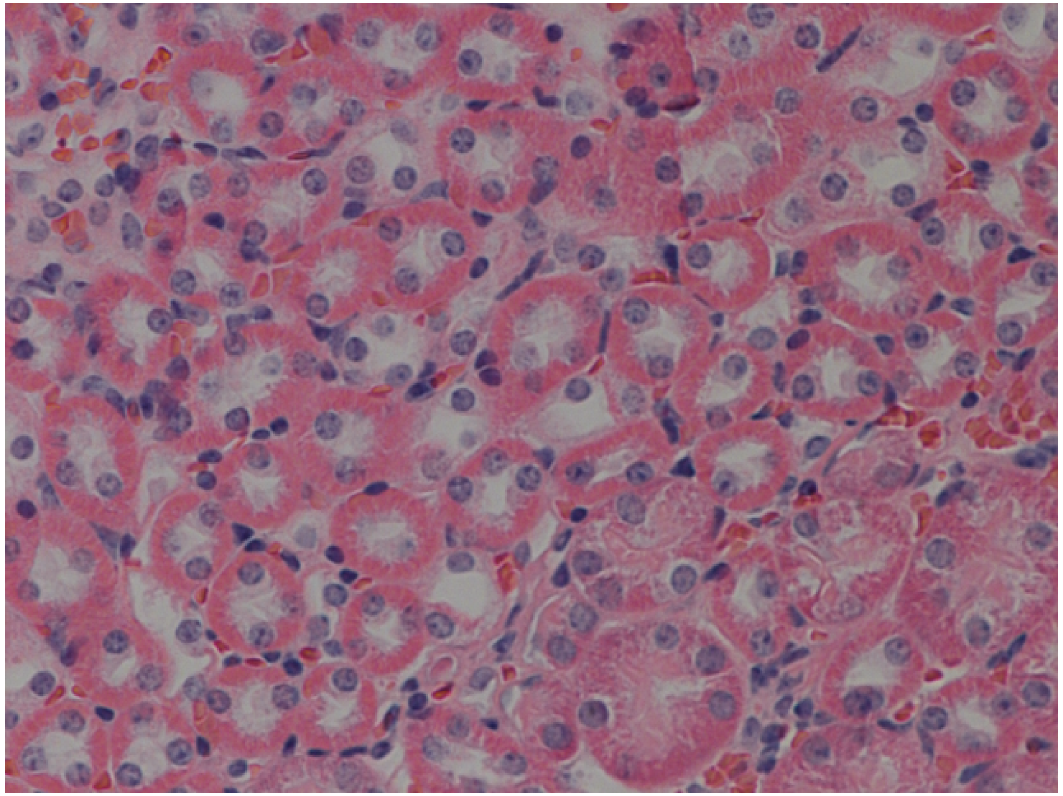

I/R Kidney Tissue

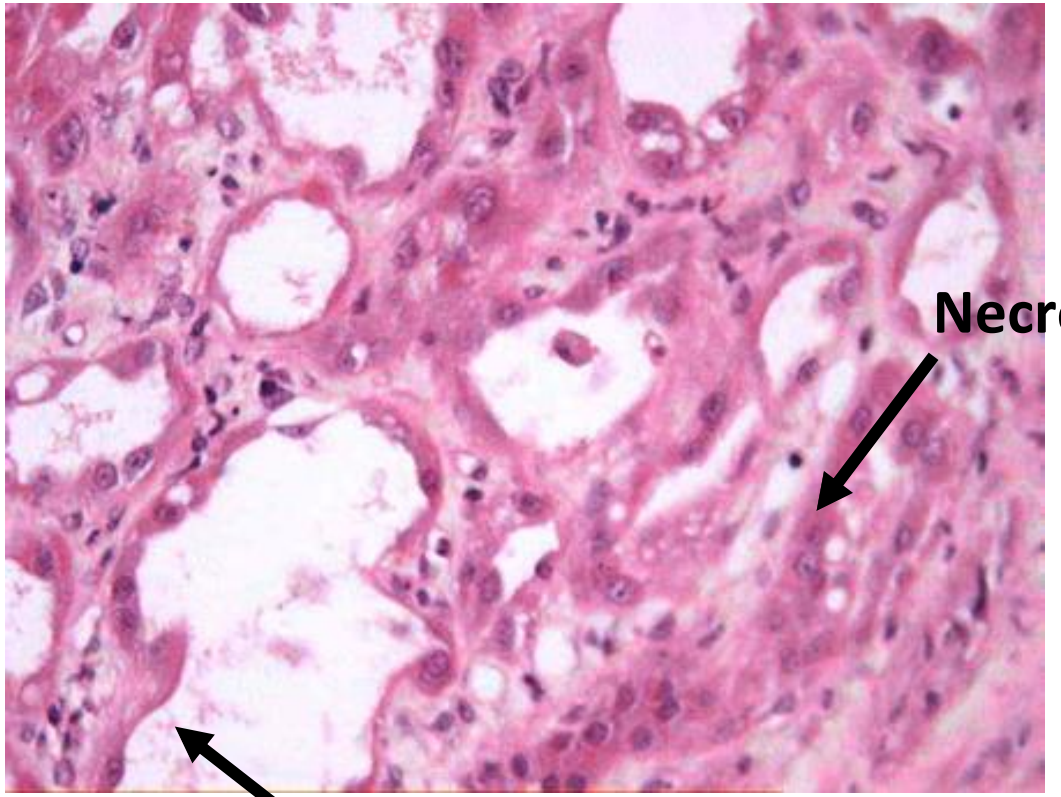

Necrosis

Loss of Brush Border

<u>Scores</u>

0 = no injury
1 = minimal (0-25%of the section)
2 = mild (25-50%)
3 = significant (50-75%)
4 = severe (more than 75%)

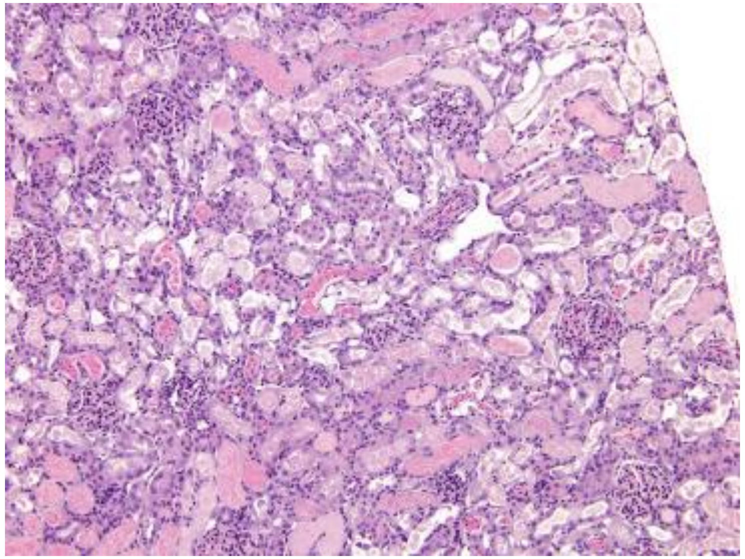

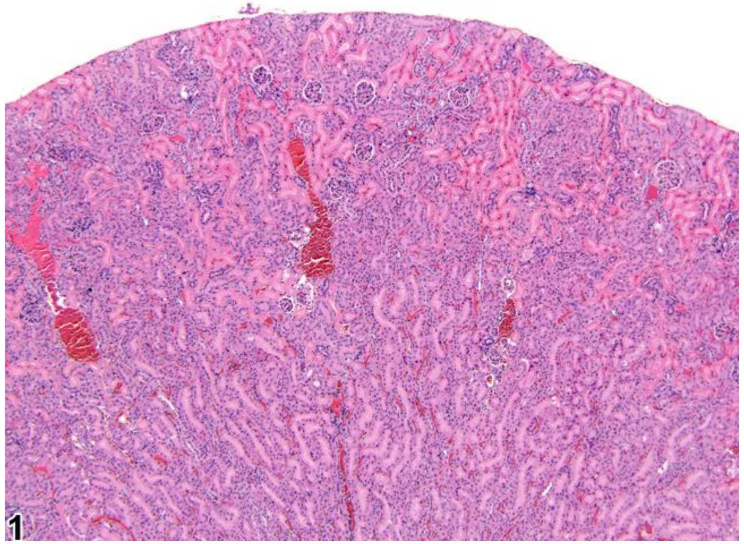

Loss of Brush Border (40X)		
Tubular Necrosis (40X)		
Neutrophil Infiltration (10X)	3	4
Hemorrhage/Congestion (10x)	1	3
Total	4	7

Scores

0 = no injury
1 = minimal (0-25%of the section)
2 = mild (25-50%)
3 = significant (50-75%)
4 = severe (more than 75%)

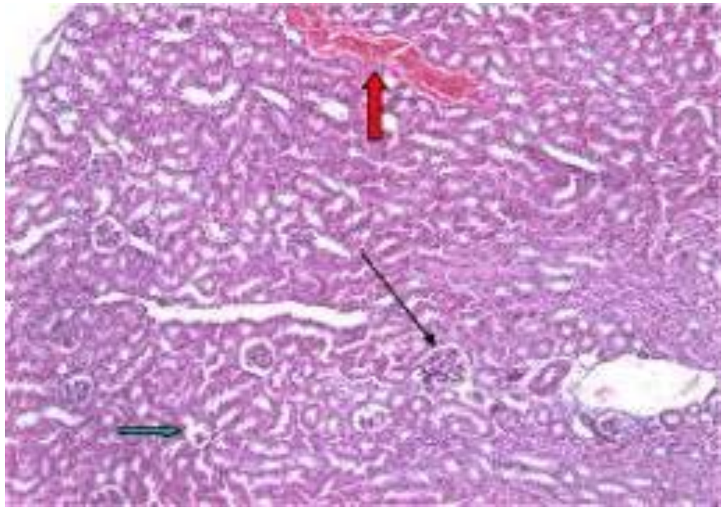

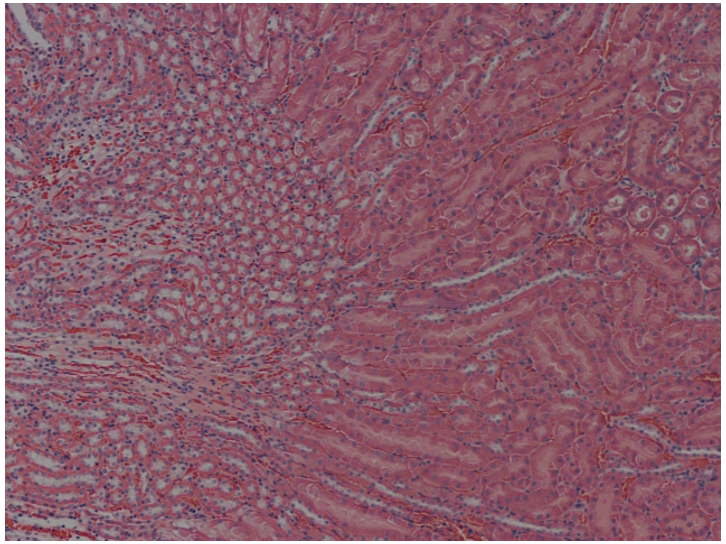

Loss of Brush Border (40X)		
Tubular Necrosis (40X)		
Neutrophil Infiltration (10X)	0	0
Hemorrhage/Congestion (10x)	1	0
Total	1	0

Adrenal Gland

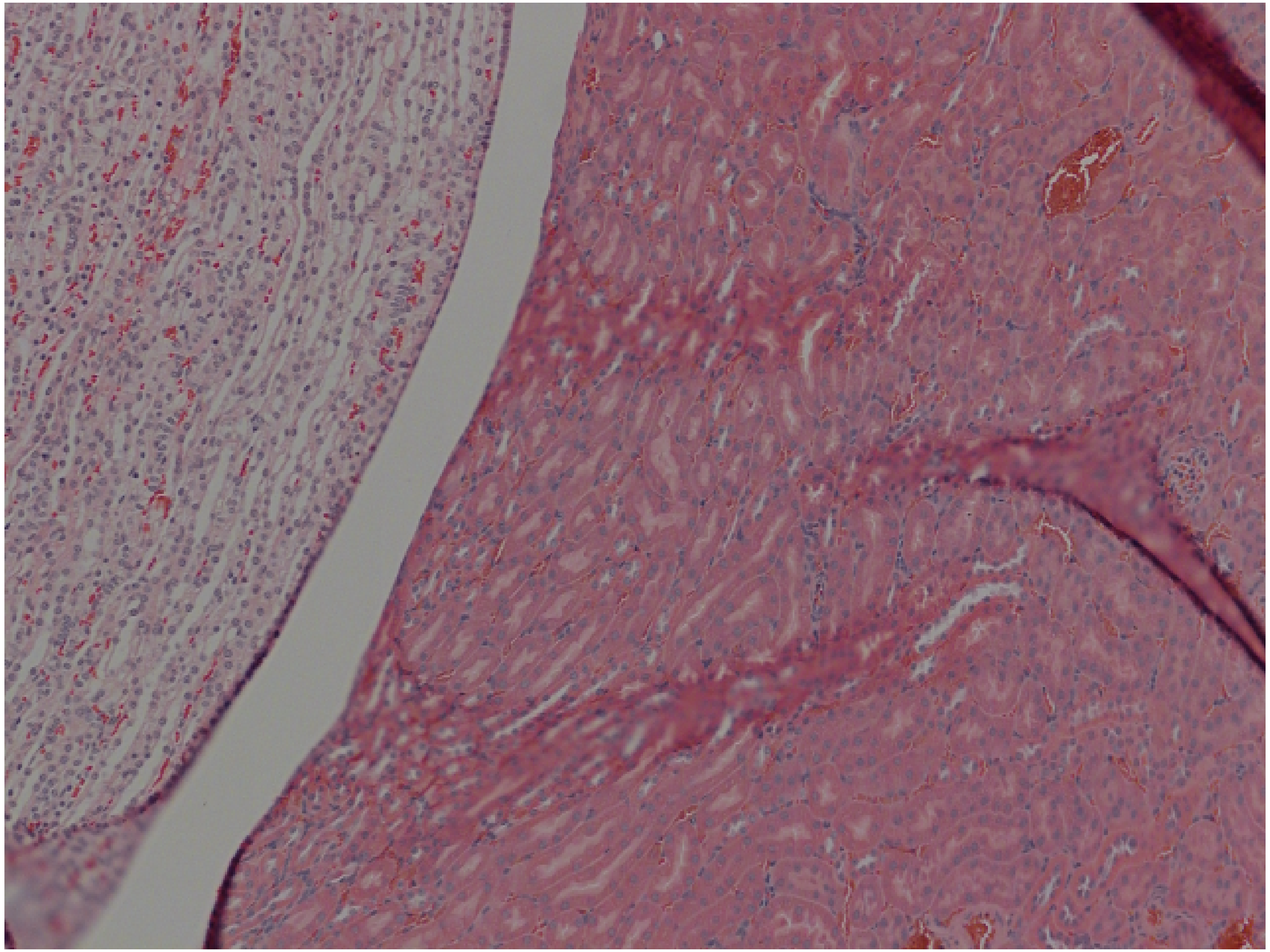

<u>Scores</u>
0 = no injury
1 = minimal (0-25%of the section)
2 = mild (25-50%)
3 = significant (50-75%)
4 = severe (more than 75%)

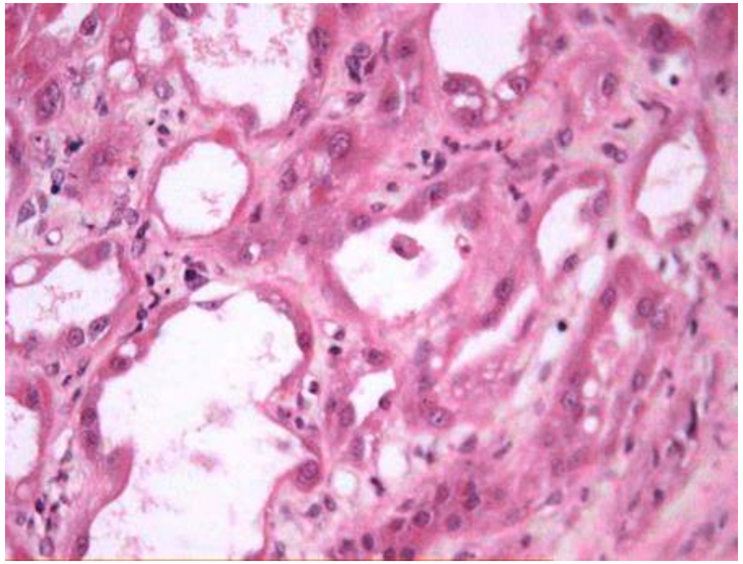

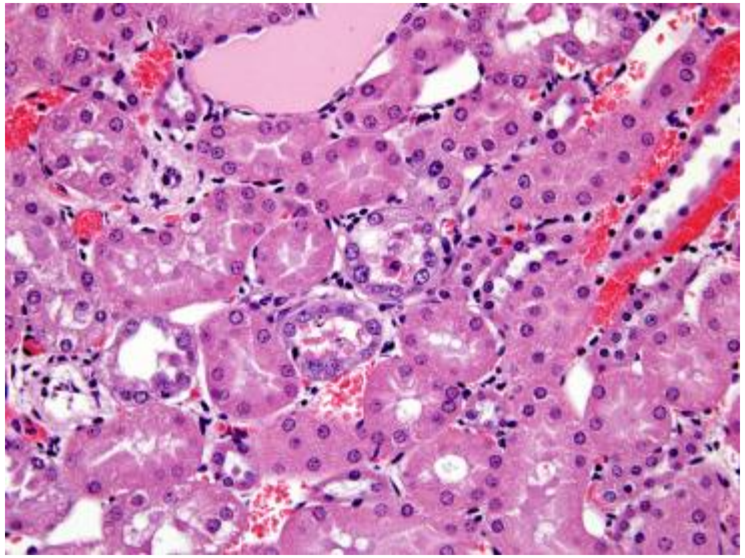

Loss of Brush Border (40X)	4	2
Tubular Necrosis (40X)	4	3
Neutrophil Infiltration (10X)		
Hemorrhage/Congestion (10x)		
Total	8	5

Scores
0 = no injury
1 = minimal (0-25%of the section)
2 = mild (25-50%)
3 = significant (50-75%)
4 = severe (more than 75%)

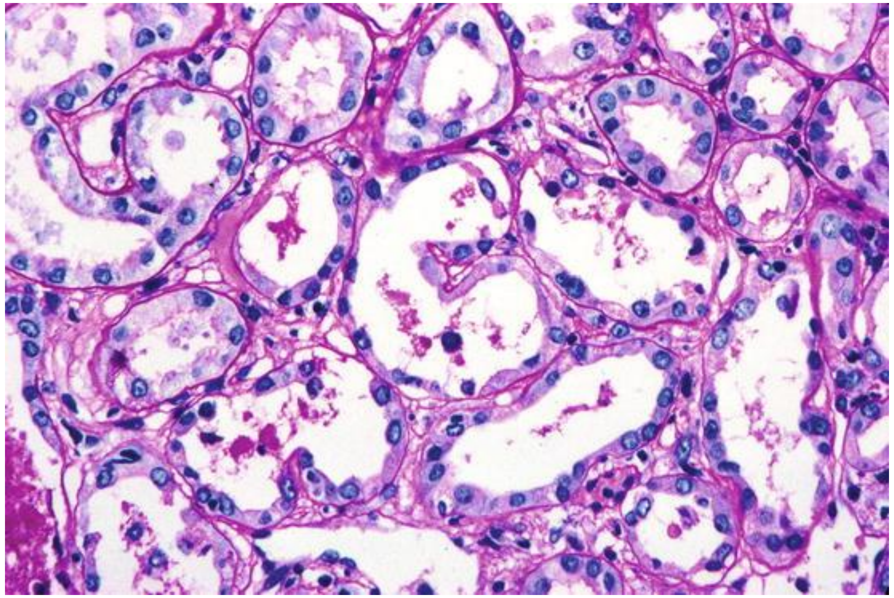

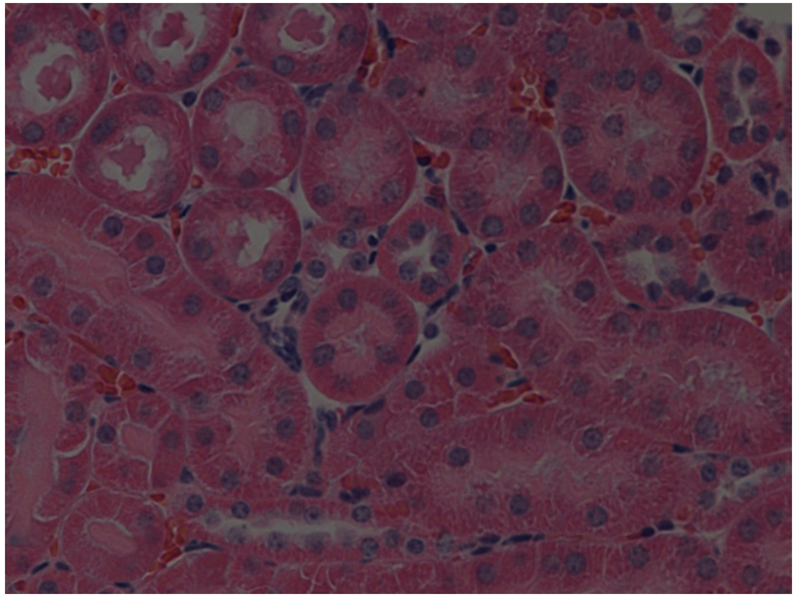

Loss of Brush Border (40X)	4	0
Tubular Necrosis (40X)	2	0
Neutrophil Infiltration (10X)		
Hemorrhage/Congestion (10x)		
Total	8	0
