# Supplementary material for: KF4 anti-CELA1 Antibody and Purified α1-Antitrypsin Have Similar but Not Additive Efficacy in Preventing Emphysema in Murine α1-Antitrypsin Deficiency
Source: bioRxiv. 2024 May 10:2024.05.07.592994. Preprint. [Version 1] doi: 10.1101/2024.05.07.592994 (PMC11100728; doi:10.1101/2024.05.07.592994)
Supplement: Supplement 3 [file media-3.pdf]

Histological Signs of Heart Injury

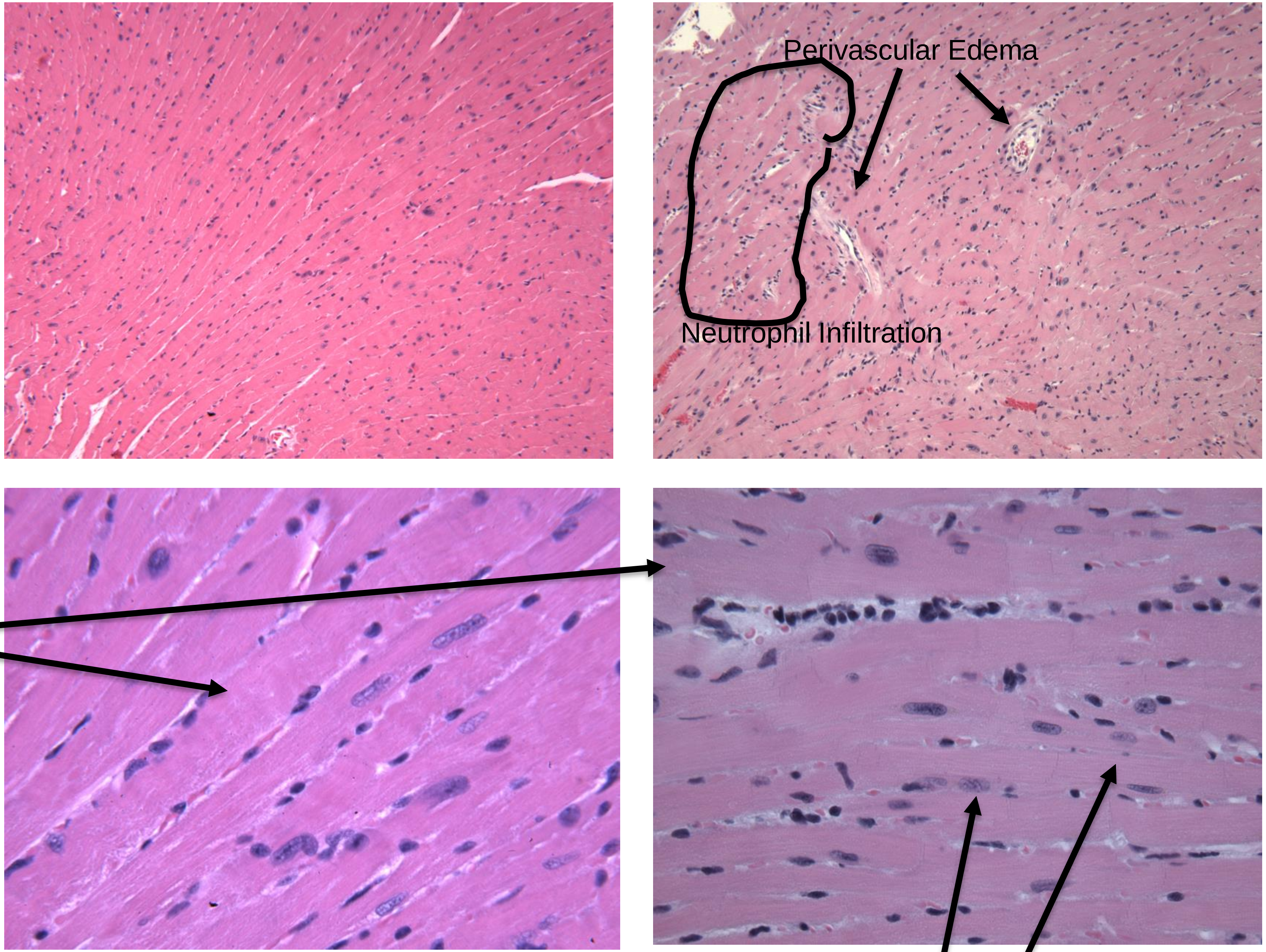

Myofibril Derangement
(fibrils with bands vs not)

Nuclear Hydrops (swollen, “ghost” nuclei)

<u>Scores</u>
0 = no injury
1 = minimal (0-25%of the section)
2 = mild (25-50%)
3 = significant (50-75%)
4 = severe (more than 75%)

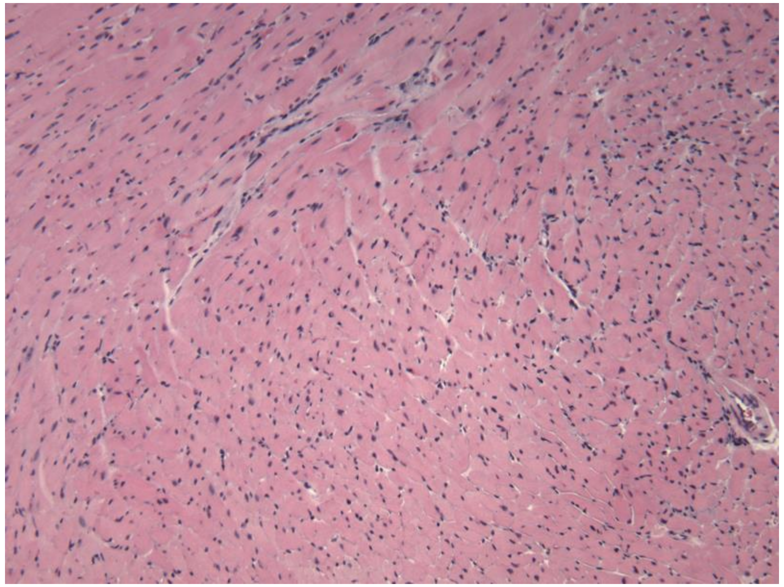

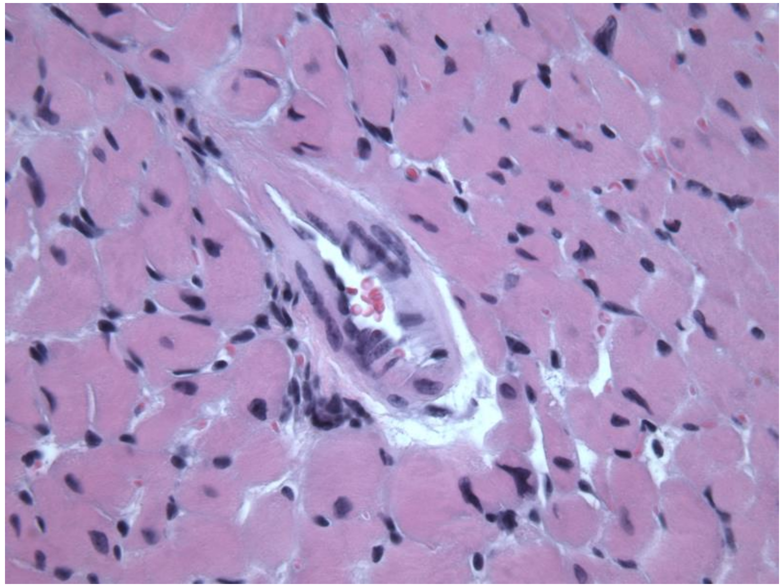

	10x	40x
Perivascular Edema (10X)	2	
Myofibril Derangement (40X)		3
Infiltration of Neutrophils (10X)	3	
Nuclear Hydrops (40X)		1
Total		

Scores
0 = no injury
1 = minimal (0-25%of the section)
2 = mild (25-50%)
3 = significant (50-75%)
4 = severe (more than 75%)

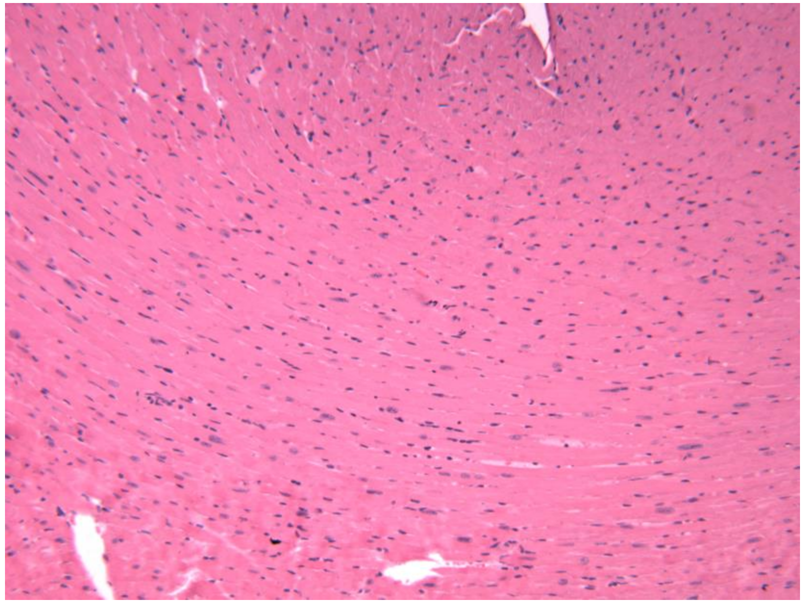

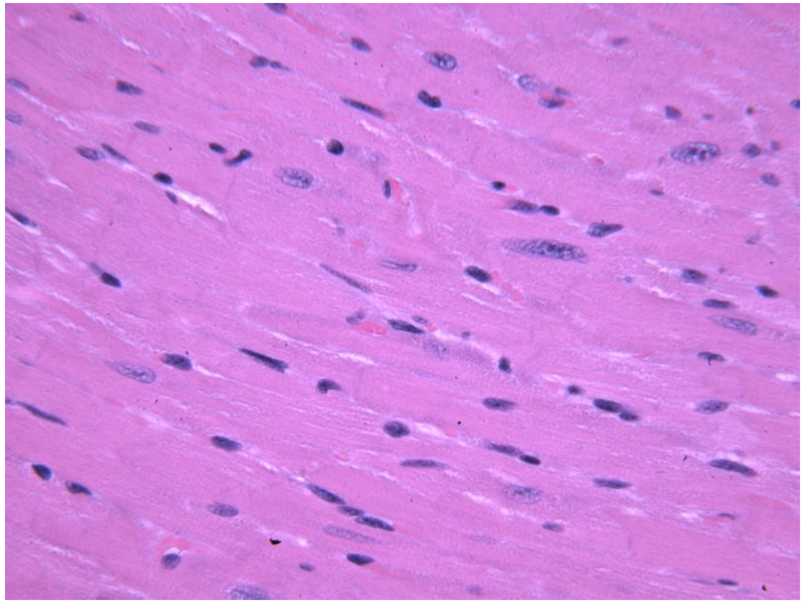

	10x	40x
Perivascular Edema (10X)	0	
Myofibril Derangement (40X)		1
Infiltration of Neutrophils (10X)	1	
Nuclear Hydrops (40X)		0
Total		

<u>Scores</u>

0 = no injury
1 = minimal (0-25%of the section)
2 = mild (25-50%)
3 = significant (50-75%)
4 = severe (more than 75%)

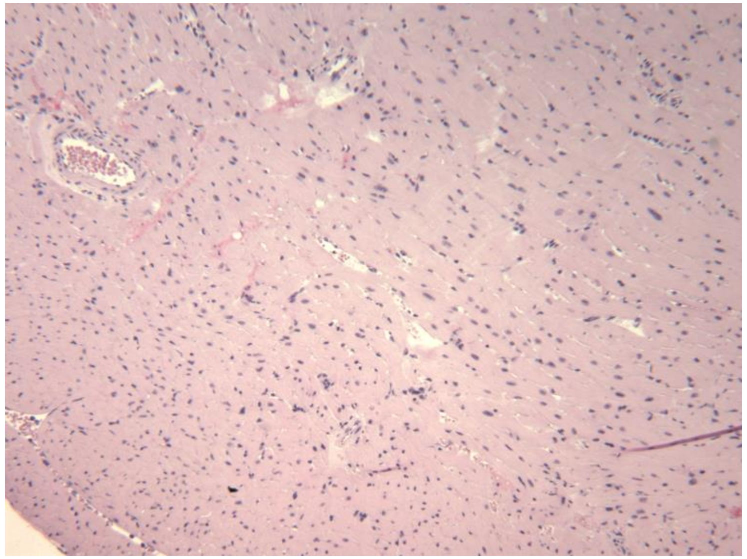

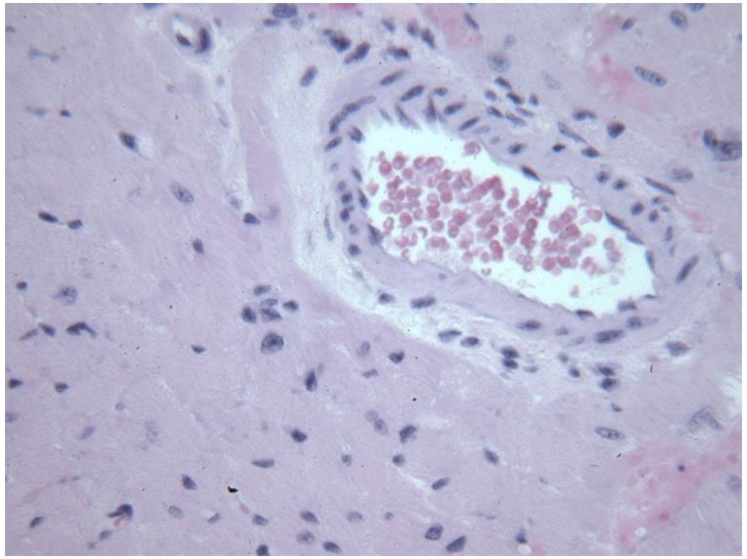

	10x	40x
Perivascular Edema (10X)	3	
Myofibril Derangement (40X)		3
Infiltration of Neutrophils (10X)	3	
Nuclear Hydrops (40X)		3
Total		

Scores
0 = no injury
1 = minimal (0-25%of the section)
2 = mild (25-50%)
3 = significant (50-75%)
4 = severe (more than 75%)

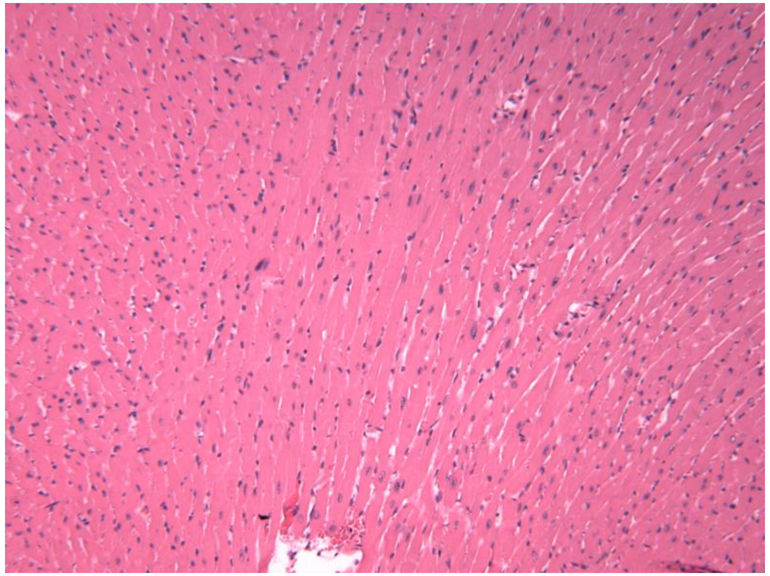

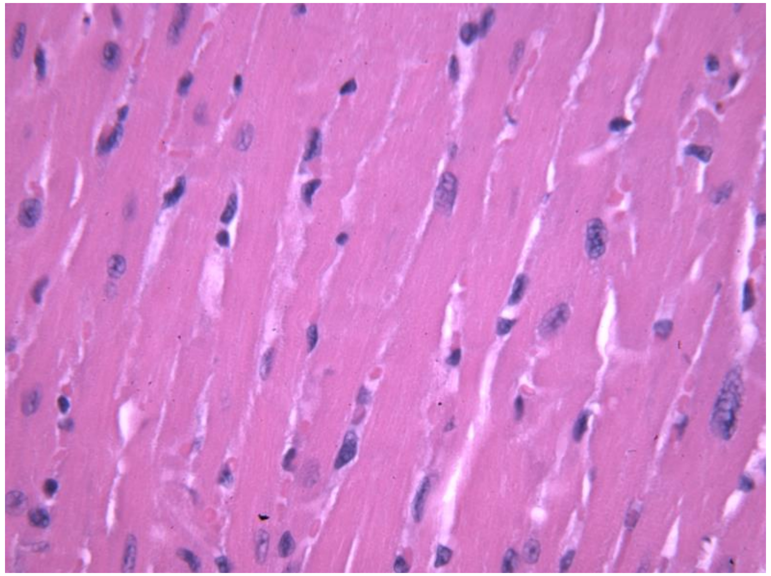

	10x	40x
Perivascular Edema (10X)	1	
Myofibril Derangement (40X)		1
Infiltration of Neutrophils (10X)	2	
Nuclear Hydrops (40X)		0
Total		
