# Supplementary material for: KF4 anti-CELA1 Antibody and Purified α1-Antitrypsin Have Similar but Not Additive Efficacy in Preventing Emphysema in Murine α1-Antitrypsin Deficiency
Source: bioRxiv. 2024 May 10:2024.05.07.592994. Preprint. [Version 1] doi: 10.1101/2024.05.07.592994 (PMC11100728; doi:10.1101/2024.05.07.592994)
Supplement: Supplement 4 [file media-4.pdf]

Histological signs of lung injury

Healthy Lung Tissue

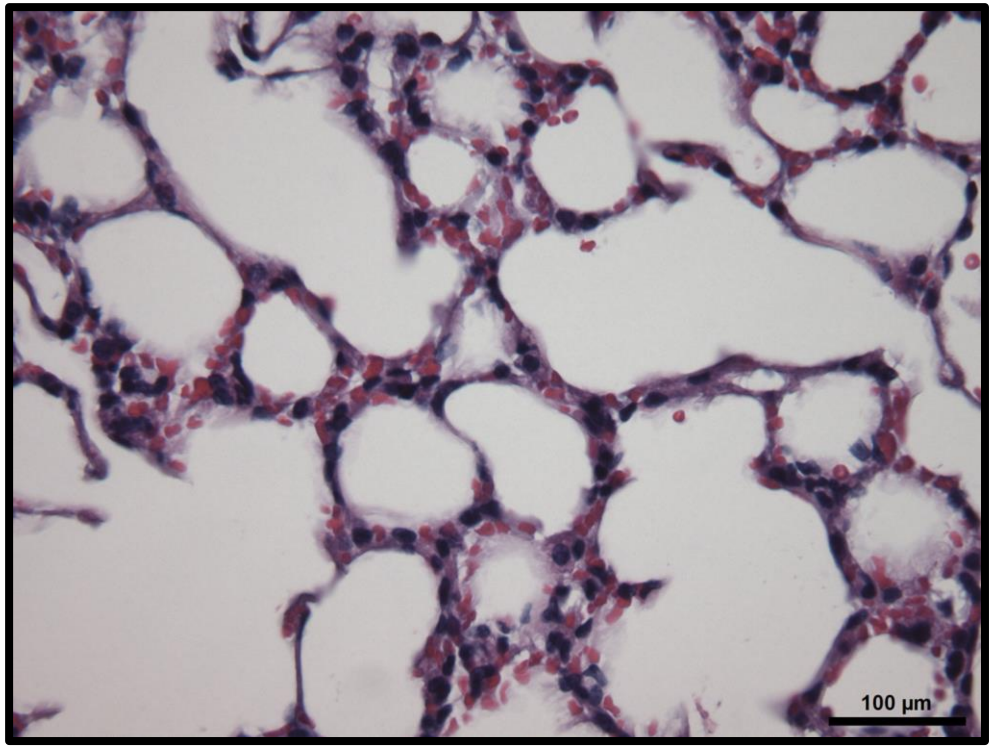

Septic Lung Tissue

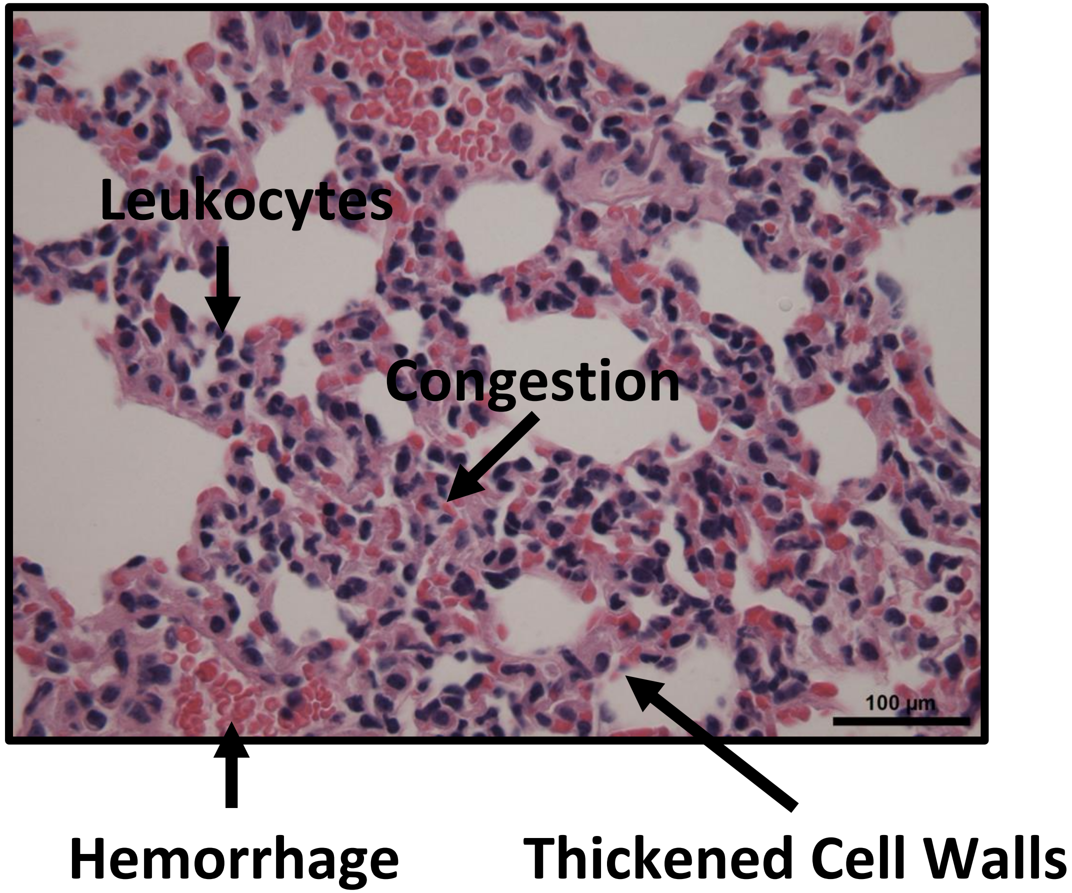

<u>Scores</u>
0 = no injury
1 = minimal (0-25%of the section)
2 = mild (25-50%)
3 = significant (50-75%)
4 = severe (more than 75%)

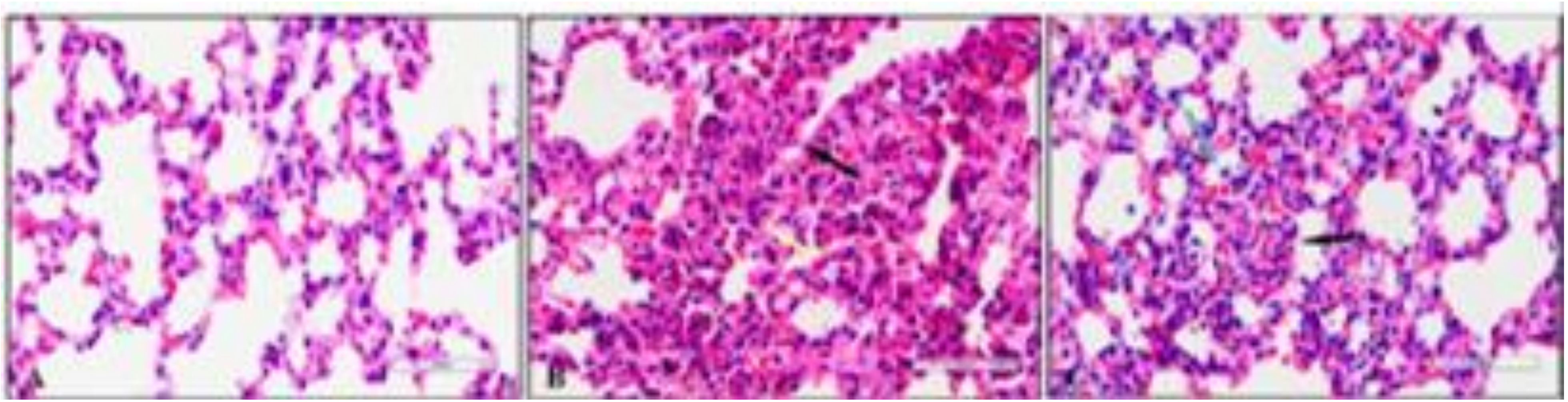

Alveolar Congestion or Reduction of Alveolar Space	1	3	2
Hemorrhage	1	3	3
Infiltration of Leukocytes into Airspace or Alveolar Walls	2	3	3
Thickness of Alveolar Wall or Hyaline Membrane Formation	2	4	3
Total	6	13	11

Scores

0 = no injury
1 = minimal (0-25%of the section)
2 = mild (25-50%)
3 = significant (50-75%)
4 = severe (more than 75%)

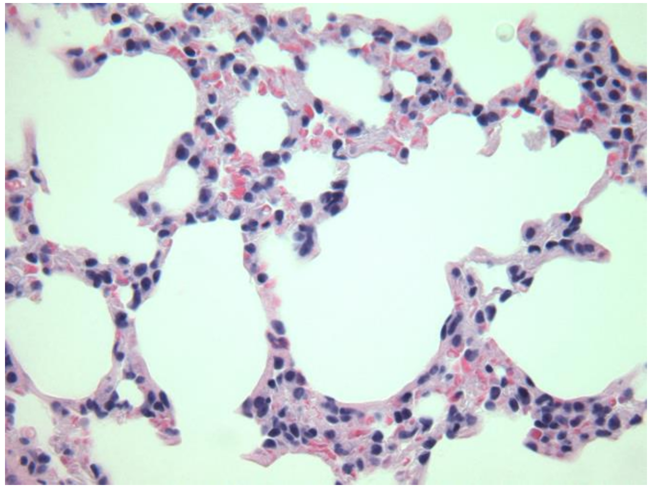

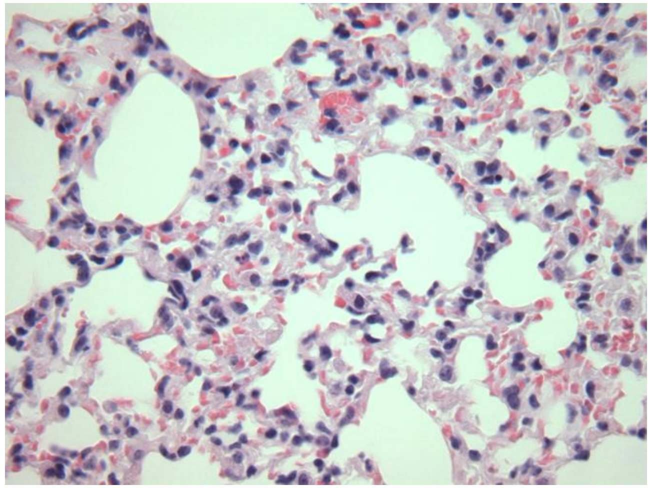

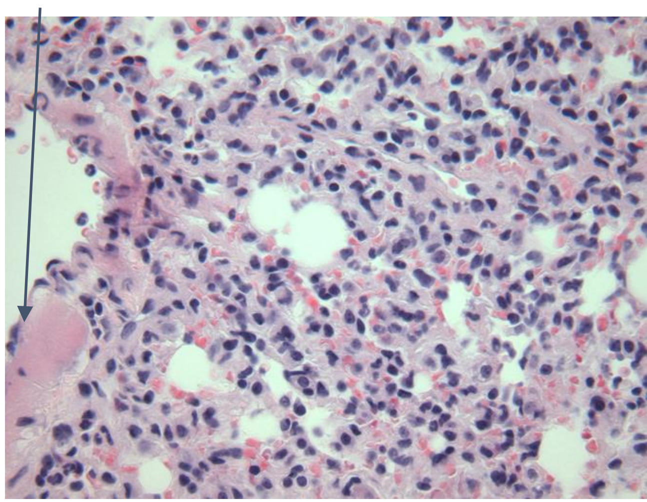

Alveolar Congestion or Reduction of Alveolar Space	1	2	3
Hemorrhage	0	1	1
Infiltration of Leukocytes into Airspace or Alveolar Walls	2	2	3
Thickness of Alveolar Wall or Hyaline Membrane Formation	1	2	4
Total	4	7	11

<u>Scores</u>
0 = no injury
1 = minimal (0-25%of the section)
2 = mild (25-50%)
3 = significant (50-75%)
4 = severe (more than 75%)

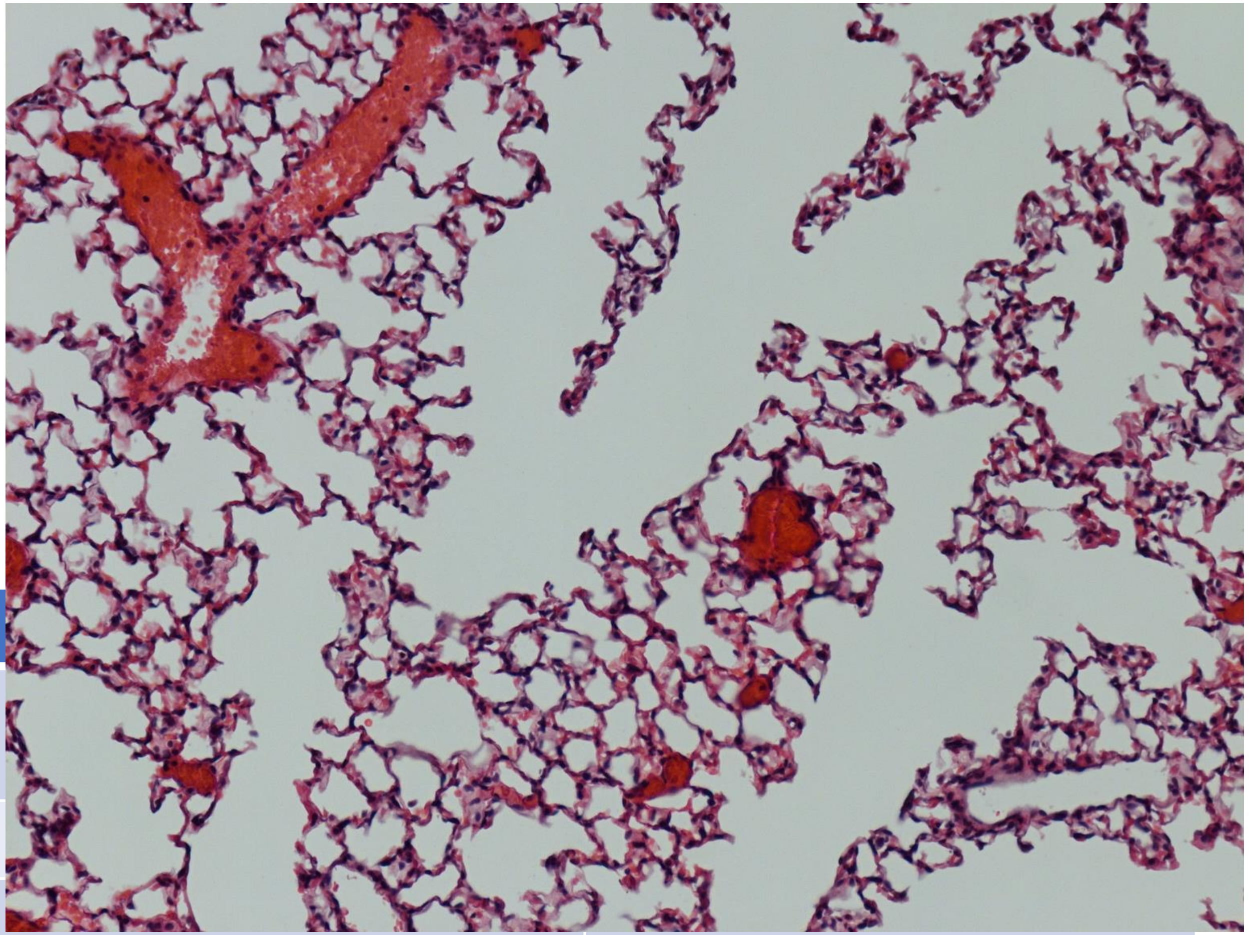

Alveolar Congestion or Reduction of Alveolar Space	0		
Hemorrhage	0		
Infiltration of Leukocytes into Airspace or Alveolar Walls	1		
Thickness of Alveolar Wall or Hyaline Membrane Formation	2 (hyaline membranes)		
Total	3		
